# Institutionalising maternal and newborn quality-of-care standards in Bangladesh, Ghana and Tanzania: a quasi-experimental study

**DOI:** 10.1136/bmjgh-2022-009471

**Published:** 2022-09-20

**Authors:** Alexander Manu, Sk Massum Billah, John Williams, Stella Kilima, Francis Yeji, Ziaul Matin, Asia Hussein, Fatima Gohar, Priscilla Wobil, Peter Baffoe, Farhana Karim, Projestine Muganyizi, Deus Mogela, Shams El Arifeen, Maya Vandenent, Kyaw Aung, Mrunal Shetye, Kim Eva Dickson, Nabila Zaka, Luwei Pearson, Tedbabe D Hailegebriel

**Affiliations:** 1 Nutrition and Public Health Interventions, London School of Hygiene and Tropical Medicine Faculty of Epidemiology and Population Health, London, UK; 2 Epidemiology and Disease Control, University of Ghana School of Public Health, Accra, Ghana; 3 Maternal and Child Health Division, ICDDRB, Dhaka, Bangladesh; 4 Maternal and Child Health Cluster, Dodowa Health Research Centre, Ghana Health Service, Accra, Ghana; 5 National Institute for Medical Research, Dar es Salaam, Tanzania; 6 Maternal and Child Health, Navrongo Health Research Centre, Navrongo, Ghana; 7 Health Section, UNICEF Bangladesh, Dhaka, Bangladesh; 8 Health Section, UNICEF, Dar es Salaam, Tanzania; 9 Maternal and Child Health, UNICEF Eastern and Southern Africa Regional Office, Nairobi, Kenya; 10 Health, UNICEF Ghana, Accra, Ghana; 11 MCHD, International Centre for Diarrhoeal Disease Research, Bangladesh, Dhaka, Bangladesh; 12 Department of Obstetrics & Gynaecology, Muhimbili University of Health and Allied Sciences (MUHAS), Dar es Salaam, Tanzania; 13 Blood Transfusion Services, Dar es Salaam, Tanzania; 14 MCHD, ICDDR,B, Dhaka, Bangladesh; 15 Health Section, UNICEF, New York, New York, USA; 16 Health Services Academy, Islamabad, Pakistan; 17 Health, UNICEF, Briarcliff Manor, New York, USA

**Keywords:** child health, health policy, public health, health systems, health services research

## Abstract

**Introduction:**

Facility interventions to improve quality of care around childbirth are known but need to be packaged, tested and institutionalised within health systems to impact on maternal and newborn outcomes.

**Methods:**

We conducted cross-sectional assessments at baseline (2016) and after 18 months of provider-led implementation of UNICEF/WHO’s Every Mother Every Newborn Quality Improvement (EMEN-QI) standards (preceding the WHO Standards for improving quality of maternal and newborn care in health facilities). 19 hospitals and health centres (2.8M catchment population) in Bangladesh, Ghana and Tanzania were involved and 24 from adjoining districts served for ‘comparison’. We interviewed 43 facility managers and 818 providers, observed 1516 client–provider interactions, reviewed 12 020 records and exit-interviewed 1826 newly delivered women. We computed a 39-criteria institutionalisation score combining clinical, patient rights and cross-cutting domains from EMEN-QI and used routine/District Health Information System V.2 data to assess the impact on perinatal and maternal mortality.

**Results:**

EMEN-QI standards institutionalisation score increased from 61% to 80% during EMEN-QI implementation, exceeding 75% target. All mortality indicators showed a downward trajectory though not all reached statistical significance. Newborn case-fatality rate fell significantly by 25% in Bangladesh (RR=0·75 (95% CI=0·59 to 0·96), p=0·017) and 85% in Tanzania (RR=0.15 (95% CI=0.08 to 0.29), p<0.001), but not in Ghana. Similarly, stillbirth (RR=0.64 (95% CI=0.45 to 0.92), p<0.01) and perinatal mortality in Tanzania reduced significantly (RR=0.59 (95% CI=0.40 to 0.87), p=0.007). Institutional maternal mortality ratios generally reduced but were only significant in Ghana: 362/100 000 to 207/100 000 livebirths (RR=0.57 (95% CI=0.33 to 0.99), p=0.046). Routine mortality data from comparison facilities were limited and scarce. Systematic death audits and clinical mentorship drove these achievements but challenges still remain around human resource management and equipment maintenance systems.

**Conclusion:**

Institutionalisation of the UNICEF/WHO EMEN-QI standards as a package is feasible within existing health systems and may reduce mortality around childbirth. Critical gaps around sustainability must be fundamental considerations for scale-up.

**WHAT IS ALREADY KNOWN ON THIS TOPIC**

- Quality improvement (QI) interventions are demonstrating improvements in provider practices in many settings but, in controlled studies, these impovements have not been found to be significantly different from practices in control settings.
- Studies showing mortality effects of QI interventions are lacking leading to perceptions that QI interventions that will impact on mortality may not be feasible to implement, as a package, within existing health systems.

**WHAT THIS STUDY ADDS**

- After just 18 months of Every Mother Every Newborn Quality Improvement (EMEN-QI) implementation within existing health systems, significant mortality reductions were acheived: facility newborn case fatality rate fell significantly by 25% in Bangladesh (p=0·017) and 85% in Tanzania (p<0.01); stillbirth rate fell by significantly by 36% (p=0·001) and perinatal mortality rate by 41% (p=0·007) in Tanzania whilst maternal mortality ratio fell in Ghana by 43% (p=0.046).
- The mortality effects (driven mainly by the devolution of improved quality maternal and newborn care to the district level and augmenting these with mortality audits and actions on recommendations) were matched by increased uptake of EMEN-QI standards with the institutionalisation composite uptake index increasing from 61% to 80% across the countries, well above the target set *a priori* at 75%.

**HOW THIS STUDY MIGHT AFFECT RESEARCH, PRACTICE OR POLICY**

- WHO’s standards for improving quality of maternal and newborn care in health facilities (modelled from the EMEN-QI standards) are feasible to implement, as a package, within existing health systems in low-income and middle-income countries, can lead to improved provider care practices, and within the right policy environment inputs, can impact on maternal and perinatal mortality.
- The next critical step is the development of easy-to-measure (in order not to burden providers) metrics around implementation using studies with more robust designs, matched with the development of local capacity to use the data for decision-making as being provided on the WHO Quality Equity Dignity network platform.

## Introduction

 The Lancet Quality Commission proposed that efficiency, equity, people centredness and resilience in the delivery of quality care within health systems should be core values if quality improvement (QI) must serve as an entry point for system-wide improvements to ensure universal access to care and address the quality needs of the most vulnerable at the riskiest times.^1 2^ Substantial progress has been made in maternal and child survival; global maternal deaths have reduced from 532 000 in 1990 to 295 000 in 2017^3^ and child mortality from 12·6 million in 1990 to 5.3 million in 2018.^4^ Antenatal clinic attendance is over 90% and more than 70% of women currently deliver in health facilities.^5^ However, in 2018, the 2·6 million stillbirths and 2·5 million neonatal deaths can be attributed to poor quality of obstetric and newborn care. The American Academies of Science and Medicine report of 2018 estimated that 462 000 of the 2·5 million neonatal deaths and 44 000 of 278 000 maternal deaths in 2015 were related to poor quality of care.^6^ Bridging the quality gap is therefore key to reducing mortality and improving health outcomes, to achieve universal coverage^7^ and to meet the global sustainable development goals.^8^,^10^

The World Health Assembly ratified the Every Newborn Action Plan (ENAP) in 2015 (Resolution WHA 67·10).^11^ UNICEF and WHO adopted Every Mother Every Newborn Quality Improvement (EMEN-QI) initiative as a strategy for implementation. UNICEF/WHO jointly developed ten standards and set criteria aimed at improving the quality of facility maternal and newborn care along three domains—clinical care, patients’ rights and cross-cutting issues. These standards were later translated into the 2016 eight Standards for improving quality of maternal and newborn care in health facilities^12^ to reduce mortality and severe morbidity although they were not tested, as a package, in any low-income and middle-income country (LMIC).

We took advantage of an ongoing Mother and Baby Friendly Hospital Initiative to pilot the EMEN-QI standards at 59 health facilities in Bangladesh, Ghana and Tanzania through a collaboration between the Ministries of Health and UNICEF headquarters/country offices. We hypothesised that institutionalising EMEN-QI package is feasible within health systems of LMICs, will improve the provision and experience of care as well as have a positive impact on perinatal outcomes.^13^ The lessons learnt would also inform the global roll-out of WHO’s quality standards, exemplified in WHO/UNICEF’s Quality Equity Dignity network.^14^

## Methods

### Overview of the EMEN-QI implementation

#### Setting

EMEN-QI was implemented in seven districts (2·8 million population) across the three countries (online supplemental table S1)—2 069 273 in Kurigram (Rangpur division, Bangladesh)^15^; 391 563 in Bawku, Bolgatanga Bongo and Kassena-Nankana West (Upper East region, Ghana)^16^ and 373 740 in Ludewa and Wangin’gombe (Njombe region, Tanzania).^17^ These were UNICEF-focused districts (ie, districts where UNICEF has existing presence or programming) with either low numbers of health interventions (Kurigram, Bangladesh), social disadvantages (Upper East, Ghana) or poor maternal and newborn health (MNH) indicators (Njombe and Rangpur).

The 59 intervention facilities included 5 in Bangladesh, 24 in Ghana and 20 in Tanzania. The primary focus was on 43 district/municipal/regional hospitals and subdistrict health centres. However, to develop a district-wide QI model, Ghana and Tanzania involved 16 arterial community clinics and dispensaries. The conceptualisation was to align implementation with national priorities in the respective countries and also ensure safeguarding for participants and the evaluation team.

#### Interventions

Table 1 shows the key interventions implemented as highlighted in the following:

- Inputs and processes: development of infrastructure to support the provision of quality care for mothers and newborns followed by QI processes establishment and institutionalisation.

**Table 1:** Interventions implemented in facilities within the three countries implementing EMEN-QI

Targeted intervention	Priority interventions implemented in EMEN-QI	Country(ies)
Development of infrastructure to support the provision of quality care for mothers and newborns	1. Construction of new facilities and renovation of existing ones	TZ
	2. Upgrading of existing facilities to provide added functions, for example, theatres in health centres for Comprehensive Emergency Obstetric Care (CEmOC) services and/or increasing bed capacity	BD, TZ
	3. Construction of toilet and bathing facilities in health centres	GH
	4. Construction of mechanised boreholes for water supply	GH
	5. Establishment of special care newborn units in existing facilities	All countries
	6. Establishment of newborn stabilisation unit in existing facilities	BD
	7. Establishment of Kangaroo mother care (KMC) units	All countries
	8. Procurement of motorbikes to support outreach and postnatal home visits	GH
	9. Procurement of equipment, drugs and supplies	All countries
Establishment and institutionalisation of QI processes	10. Formation and improving functionality of QI teams	All countries
	11. Support for training and placement of human resources for health to facilitate QI	BD, TZ
	12. Training, mentorship and coaching support (on site)	All countries
	13. Introduction and strengthening maternal and newborn health (and breastfeeding) counselling	All countries
	14. Death review and response (maternal and perinatal)	All countries
	15. Periodic external supervision by (sub)national team	All countries
	16. Strengthening Health Management Information System to incorporate quality indicators as per EMEN and creation of Dashboards in the District Health Information System V.2	All countries
	17. Strengthening national level oversight	All countries
Implementation of QI interventions around the provision and experience of care	18. Infection prevention and control	All countries
	19. Water, sanitation and hygiene in health facilities	All countries
	20. Adaptation, adoption and use of QI protocols for caregiving	All countries
	21. Working-area organisation to facilitate care provision including the sort, set in order, shine, standardise, sustain	All countries
	22. Effective triaging in maternity care	All countries
	23. Ensuring privacy in caregiving	All countries
	24. Effective labour monitoring using partographs	All countries
	25. Postnatal care and counselling	All countries
	26. Newborn care: resuscitation, breastfeeding promotion, treatment of infections, warm chain (including KMC), sick newborn care	All countries
	27. Timely referral for appropriate care	All countries
	28.Community engagement for demand-side perspectives and experience of care, including breastfeeding counselling	GH, TZ

BD, Bangladesh; EMEN-QI, Every Mother Every Newborn Quality Improvement; GH, Ghana; TZ, Tanzania.

Implementation followed a 3-month development period from October 2016, with the formation and training of QI teams within facilities. Priority interventions were identified and specific investments made. For instance, a new facility building was constructed in Wanging’ombe, Tanzania. All facilities were supported to procure critical equipment for newborn care. In Ghana, motorbikes were procured to support antenatal and postnatal outreach services and community follow-up of discharged sick and small babies.

Special care newborn units (SCNUs) and Kangaroo mother care units were established to devolve small and sick newborn care to the district level. Facility teams worked with district-level, regional-level and national-level teams to plan the implementation, developed budgets and secured funding for implementation with UNICEF’s technical assistance. For instance, clinical mentors’ accommodation was arranged locally by facility to regional-level managers with some financial/technical support from UNICEF. Human resources for health (HRH) were developed for these units through a staged process: paediatricians from teaching hospitals trained local physicians and followed-up with mentorship and support that involved cycles of 5–10 days on-site visits and use of off-site social media platforms, for example, WhatsApp/Skype. No special incentives were given to health workers as the implementation was part of their routine service delivery.

QI institutionalisation was deployed in 3-monthly cycles: QI teams consulted with district facility staff to identify ‘change ideas’ based on systematic gaps in quality care provision. They set objectives based on criteria from EMEN-QI standards, designed activities and developed metrics to monitor progress in Plan–Do–Study–Act (PDSA) cycles. In PDSA, QI teams identify a change idea, plan on how to tackle it (Plan), implement their plan (Do), assess whether the implementation has yielded the required change (Study) and, based on the findings, review and refinement the plan (Act).

- Implementation of QI interventions around the provision and experience of care

Teams in facilities initially prioritised interventions that did not require substantial funding, for example, hand washing as part of improving water, sanitation and hygiene in health facilities, infection prevention practices and work-area organisation using the 5Ss model (five Japanese words seiri, seiton, seiso, seiketsu and shitsuke, described by Hiroma^18^ that stand for ‘sort, set in order, shine, standardise and sustain’). Later on, maternal and perinatal death surveillance and response (MPDSR) systems were introduced to identify, collect and analyse data on any maternal and perinatal deaths so that providers can learn from systematic failures in the process of care giving that contributed to the death. They then make recommendations to remedy the situation and prevent future occurrence.

These recommendations became ‘change ideas’ for the QI process and their implementation was tracked. Innovations to monitor performance included ‘mystery participant observers’ in Tanzania where one staff member within the care team was selected per shift to secretly observe and report on hand washing practices of their colleagues. QI teams analysed the data, reported prevailing practices, discussed solutions to address suboptimal practices and reoriented/trained staff on recommended practices/behaviours.

Separate modules were implemented to improve routine health information management systems. External monitors visited and independently assessed facilities’ performance at each quarter’s end to guide the next step of the implementation.

### Evaluation of the EMEN-QI implementation

We used a quasiexperimental design that involved two cross-sectional assessments of hospitals and health centres at baseline (July–September 2016) and a planned follow-on assessment in July–November 2018—18 months after implementation started. The specific objectives were to examine for changes in provision and experience of care and MNH outcomes, institutionalisation of standards by estimating the proportion of the targeted facilities that met at least 75% of the EMEN standards and criteria at endline. Institutionalisation was calculated as percentage uptake of 39 EMEN criteria by the countries’ institutions (details in data analyses below). The associations with maternal and perinatal mortality were evaluated.

Detailed protocol for the evaluation has been published.^13^ We evaluated 19 EMEN-QI and 24 comparison facilities from adjoining districts that were similar to EMEN-QI facilities in terms of designation to provide Emergency Obstetric and Newborn Care, maternity caseload and catchment population’s sociocultural mix. In total, a 7·1M catchment population accessed these facilities—2.8M in EMEN-QI and 4·3M in comparison districts. The comparison districts were Gaibandha and Lalmonirhat in Bangladesh, Kassena-Nankana, Builsa North, Talensi and Bawku West in Ghana, and Njombe TC and Makete in Tanzania.^13^

Clinicians and social scientists (2–4 per team) from independent research institutions—International Center for Diarrhoeal Diseases Research, Bangladesh (ICDDR,B); Navrongo Health Research Centre, Ghana Health Service; and National Institute for Medical Research, Tanzania—were trained for the data collection. A UNICEF consultant (AM) facilitated the data collectors’ training in all countries to ensure common approaches and tools were used.

### Data collection and processing

Common tools were designed based on our published^13^ assessment framework. Data collectors spent 2 weeks in each facility at baseline and follow-on in both intervention and comparison facilities. They obtained clearance and interviewed facility managers on the availability of policies/guidelines/protocols and HRH for quality care delivery. They checked amenities available on maternity/newborn units including maternity toilets for women; consented and conducted 1826 exit interviews with newly delivered women on their experiences of childbirth care, and 818 midwives/staff on the care they provided. Study clinicians reviewed 12 020 women’s records covering the previous 3–6 months (according to facilities’ patient volume) to abstract data on care content and documentation. They passively observed 1516 client–provider interactions from reception to post delivery using a checklist. In Ghana and Tanzania, 39 key informant interviews were conducted to obtain programme managers’ and policy-makers’ perceptions on the implementation and pathways to its sustainability and scale-up. Routine data from District Health Information System V.2 were obtained in intervention facilities to assess mortality impacts. In the "comparison" facilities, lack of data improvement interventions meant that data quality was poor and were not usable.

Data were validated, underwent range and consistency checks, queries were generated and resolved. Cleaned data were stored on encrypted, password-protected servers in Stata format. Qualitative interviews were transcribed into Microsoft Word and coded into themes generated in accordance with the research objectives.

### Sample size considerations and data analyses

Online supplemental table S2 shows number of respondents for each assessment module. With 247 clinician observations from Tanzania, the study had 90% power (at 5% significance level) to detect a 5% (absolute) change in the prevalence of any practice with 10% uptake. This sample size was adequate even with a 10% adjustment for design effect. Percentages were estimated for uptake of interventions to determine changes between baseline and follow-on. In total, 39 criteria (Web appendix 1) selected from the 10 EMEN-QI standards-10 on clinical care (standards 1–3), 7 on patients’ rights (standard 4) and 22 on cross-cutting issues (standards 5–10) were used to construct a Composite Institutionalization Index (CII) for the implemented package. Simple unweighted mean percentages were computed and compared between baseline and follow-on. Institutional mortality ratio (iMMR, defined as all maternal deaths per 100 000 livebirths), neonatal case-fatality rate (NCFR, all neonatal deaths, including referrals in, per 1000 livebirths), stillbirth rate (iSBR) and perinatal mortality rate (iPMR) were estimated. Differences in proportions were tested using z tests. 95% CIs were constructed around estimates.

#### Adjustment for clustering

The unit of selection was the district-our cluster. We adjusted for clustering in the data because we considered that families’ care-seeking practices or health facilities’ care provision to patients in the same district were likely to be different from those in another district, even within the same country. This means that the intraclass correlation increases. The adjustment was done in two ways: first, we included a 10% adjustment for design effect in the sample size considerations for the records review and exit interviews; and second, we computed the effect estimates for each district and assessed differences using the standard errors around the marginal effects sizes per district/cluster.^19^ We also reported data separately for all three countries because of the differences in context and interpretations of the findings.

We read through the qualitative transcripts repeatedly, identified themes for coding, interpreted them within context and triangulated the findings with the quantitative data.

## Findings

### Maternal, fetal and newborn health outcomes

The quality of routine mortality data from non-intervention facilities was poor at the follow-on assessment and could not be used for the analyses. In the intervention facilities, routine data showed that between 2016 and 2017, the death rates increased followed by a fall as at 2018. For instance, in Ghana, NCFR increased from 5.8 per 1000 livebirths in 2016 to 18.0 in 2017 and then fell to 15.6 in 2018. Similarly, in Tanzania, iSBR increased from 17.0 to 22.4 per 1000 births between 2016 and 2017 and then fell to 14.4 by 2018 (table 2).

**Table 2:** Birth and mortality statistics for Every Mother Every Newborn facilities from routine data sources: 2016–2018

Bangladesh					
Event	2016*	2017	2018*		
Births	1426	–	2086		
Livebirths	1363	–	1998		
Stillbirths	63	–	88		
Neonatal deaths	114	–	124		
Perinatal deaths	**No early neonatal death data**				
Maternal deaths	3	–	1		
Indicator	2016		2018	RR (95% CI) _2018–2016_	P value
iSBR/1000 births	44.2	–	42.2	0.95 (0.70 to 1.31)	0.77
NCFR/1000 livebirths	83.6	–	62.9	0.75 (0.59 to 0.96)	0.02*
iPMR/1000 births	**Not applicable**				
iMMR/100 000 livebirths	220.1	–	50.1	0.23 (0.02 to 2.18)	0.16

Ghana					
Event	2016	2017	2018		
Births	9610	9390	9847		
Livebirths	9341	9122	9649		
Stillbirths	269	205	198		
Neonatal deaths	54	164	150		
Perinatal deaths	323	347	337		
Maternal deaths	21	33	20		
Indicator	2016	2017	2018	RR (95% CI) _2018–2017_	P value
iSBR/1000 births	28.0	22.7	20.1	0.92 (0.76 to 1.12)†	0.40
NCFR/1000 livebirths	5.8	18.0	15.6	0.86 (0.69 to 1.08)†	0.19
iPMR/1000 births	33.6	37.9	34.2	0.93 (0.80 to 1.07)†	0.31
iMMR/100 000 livebirths	224.8	361.6	207.3	0.57 (0.33 to 0.99)†	0.046*

Tanzania					
Event	2016*	2017	2018		
Births	1761	3304	3407		
Livebirths	1731	3230	3358		
Stillbirths	30	74	49		
Neonatal deaths	40	–	12		
Perinatal deaths	48	–	55		
Maternal deaths	2	–	3		
Indicator	2016	2017	2018	RR (95% CI) _2018–2016_	P value
iSBR/1000 births	17.0	22.4	14.4	0.64 (0.45 to 0.92)†	0.01*
NCFR/1000 livebirths	23.1	–	3.6	0.15 (0.08 to 0.29)	<0.0001*
iPMR/1000 births	27.1	–	16.1	0.59 (0.40 to 0.87)	0.01*
iMMR/100 000 livebirths	115.5	–	89.3	0.77 (0.13 to 4.62)	0.78

* Data covered July–December.

† Risk ratio between 2017 and 2018.

iMMR, institutional mortality ratio; iPMR, perinatal mortality rate; iSBR, stillbirth rate; NCFR, neonatal case-fatality rate.

**Figure 1:**
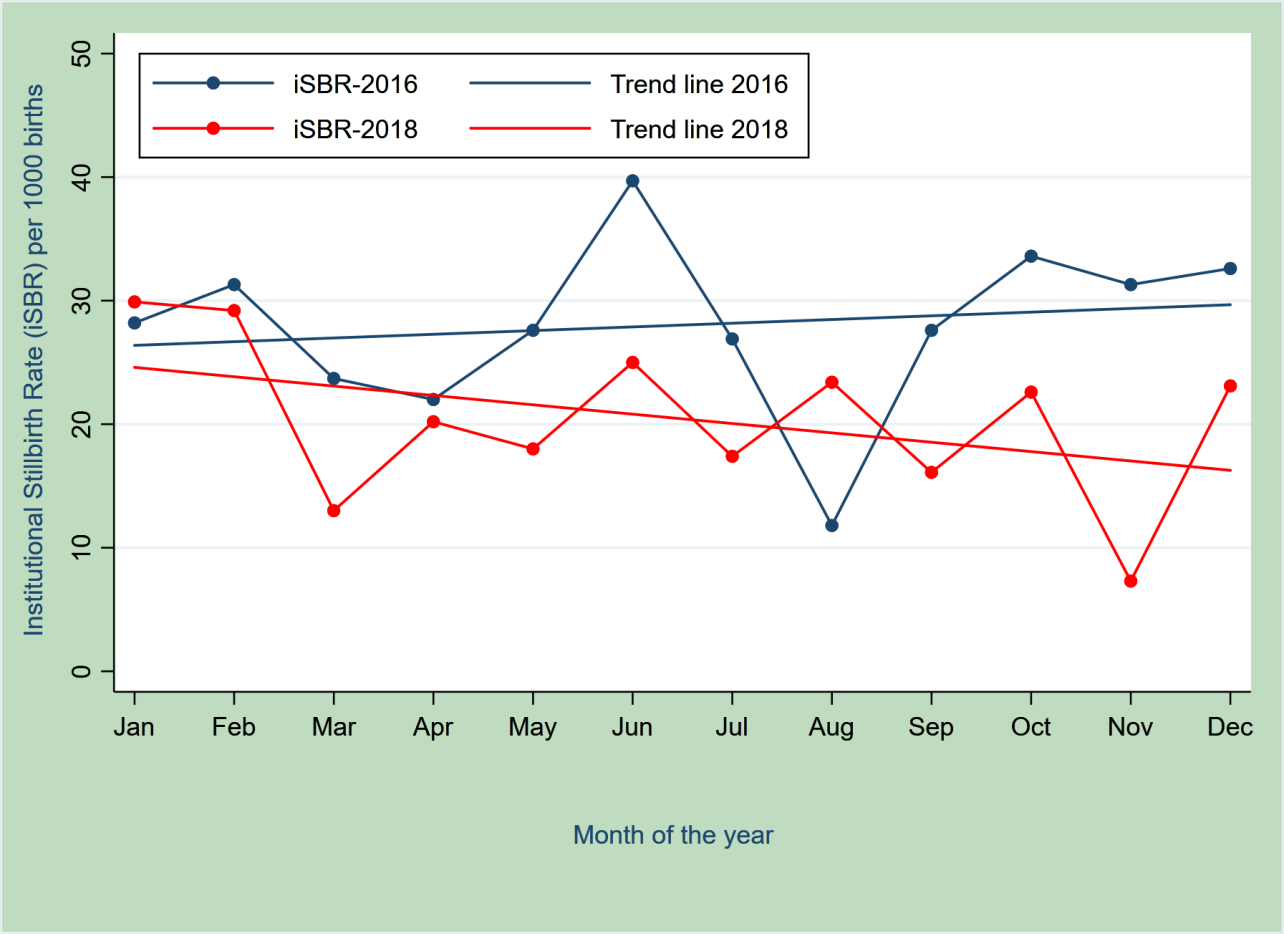
Trajectory of stillbirth rates (SBR) over the 12 months in 2016 (before Every Mother Every Newborn Quality Improvement) and 2018 (2 years later).

Table 2 also shows a consistent trend of reduction in institutional death rates across all three countries over the period. iSBR marginally reduced non-significantly in Bangladesh (5%—44·2 vs 42·2 per 1000 births—between 2016 and 2018) and Ghana (8%—22.7 vs 20·1 per 1000 births—between 2017 and 2018). In Ghana, monthly trends in iSBR for EMEN-QI facilities showed an increasing trajectory in 2016 compared with a steady fall during 2018 (figure 1). In Tanzania, however, there was a significant 36% reduction: RR=0.64 (0.45 to 0.92), p=0.01.

Overall NCFR reduced significantly from 83·6 to 62·9 per 1000 livebirths from 2016 to 2018 in Bangladesh: RR=0·75 (95% CI=0·59 to 0·96); p=0·02. Bangladesh SCANU admissions increased from 1155 to 1858 and case fatality reduced from 9·9% to 6·7% between baseline and 2018. In Ghana, NCFR fell by 14% but the data were consistent with a possible 31% reduction as well as an 8% increase in NCFR: RR=0.86 (0.69 to 1.08); p=0.19. In Tanzania, NCFR fell by a significant 85% from 23.1 in 2016 to 3.6 in 2018: RR=0.15 (0.08 to 0.29), p<0.0001.

IPMR also fell in all countries but reached statistical significance in only Tanzania—27.1 in 2016 to 16.1 in 2018: RR=0.59 (0.40 to 0.87); p=0.01. Similarly, though iMMR (per 100 000 livebirths) fell in all three countries: Bangladesh (220.1 vs 50.1), Tanzania (115.5 vs 89.3) and Ghana (362 vs 207) between baseline and 2018, it was only in Ghana that the fall reached statistical significance: RR=0.57 (0.33 to 0.99); p=0.046.

### Institutionalisation of EMEN-QI standards

**Figure 2:**
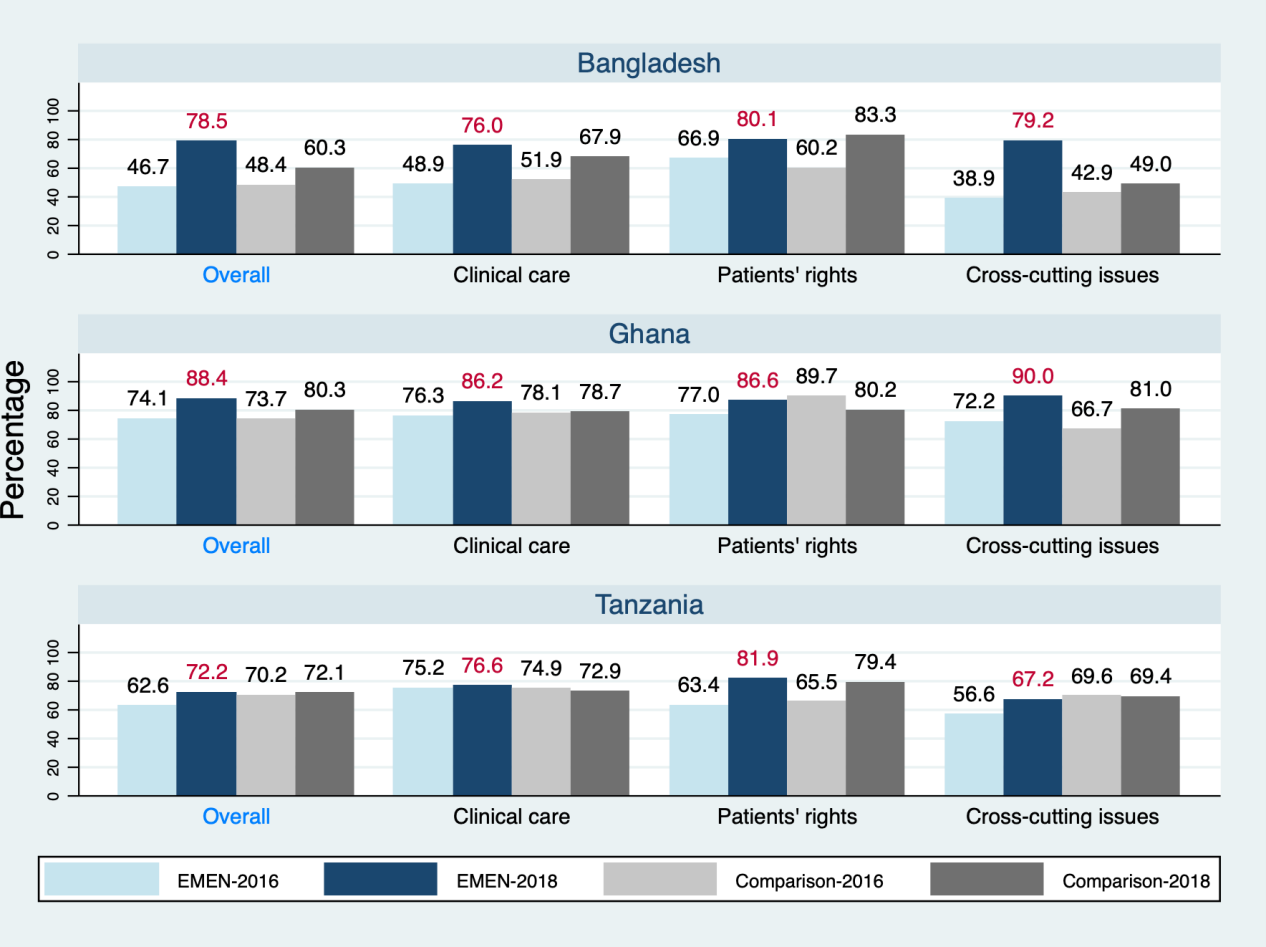
Scores of facilities in the three countries on the Composite Institutionalization Index overall and for the individual components of EMEN-QI.

The CII (table 3 and figure 2) shows that EMEN-QI facilities achieved 80% uptake of the EMEN-QI package of standards. This was a statistically significant 18% increase over the baseline (p<0.001). The trend was the same for the three domains separately: a significant increase of 13% for clinical care, 14% for patients’ rights and 23% for cross-cutting issues (p values were less than 0.01). The EMEN target of 75% institutionalisation of the standards was exceeded in all three domains as we found institutionalisation ranged from 79% to 83% at follow-on (2018) from 56% to 69% at baseline (in 2016). In comparison facilities, the CII was 71% in 2018. Only the uptake of standards around patients’ rights exceeded the 75% mark.

**Table 3:** Composite score on the institutionalisation of the three domains: clinical care, patients’ rights and cross-cutting issues of EMEN-QI standards (clinical, patients’ rights and cross-cutting issues), by country

Country	Assessment arm	Evaluation period	Performance on the Composite Institutionalization Score (number of criteria used)			
			Clinical care (10 criteria)	Patients’ rights (7 criteria)	Cross-cutting issues (22 criteria)	Overall (39 criteria)
Bangladesh	EMEN-QI	Baseline—2016	48.9%	66.9%	38.9%	46.7%
		Follow-on—2018	76.0%	80.1%	79.2%	78.5%
		Change (p value)	27.1 (p=0.016)	13.2 (p=0.20)	40.3 (p<0.001)	31.8 (p<0.001)
	Comparison	Baseline—2016	51.9%	60.2%	42.9%	48.4%
		Follow-on—2018	67.9%	83.3%	49.0%	60.3%
Ghana	EMEN-QI	Baseline—2016	76.3%	77.0%	72.2%	74.1%
		Follow-on—2018	86.2%	86.6%	90.0%	88.4%
		Change (p value)	9.9 (p=0.12)	9.5 (p=0.007)	17.8 (p=0.001)	14.3 (p<0.0001)
	Comparison	Baseline—2016	78.1%	89.7%	66.7%	73.7%
		Follow-on—2018	78.7%	80.2%	81.0%	80.3%
Tanzania	EMEN-QI	Baseline—2016	75.2%	63.4%	56.6%	62.6%
		Follow-on—2018	76.6%	81.9%	67.2%	72.2%
		Change (p value)	1.4 (p=0.74)	18.5 (p=0.012)	10.6 (p=0.11)	9.6 (p=0.017)
	Comparison	Baseline—2016	74.9%	65.5%	69.6%	70.0%
		Follow-on—2018	72.9%	79.4%	69.4%	72.1%
Overall	EMEN-QI	Baseline—2016	66.8%	69.1%	56.2%	61.3%
		Follow-on—2018	79.6%	82.8%	78.8%	79.7%
		Change (p value)	12.8 (p=0.005)	13.7 (p<0.001)	22.6 (p<0.001)	18.4 (p<0.001)
	Comparison	Baseline—2016	68.3%	71.8%	59.7%	64.1%
		Follow-on—2018	73.2%	81.0%	66.5%	70.9%

EMEN-QI, Every Mother Every Newborn Quality Improvement.

There were between-country differences—all countries saw significant increases in the CII. Overall, at the follow-on (2018), Bangladesh recorded a CII of 78.5%, a 32% jump from baseline (p<0.0001); Ghana recorded 88.4%, a 14% increase from baseline (p<0.0001); while Tanzania recorded 72.2%, indicating a significant 10% increase from baseline (p=0.017). Tanzania, however, did not attain the 75% EMEN institutionalisation target. In Bangladesh, the biggest jump in uptake of standards was the 40% increase for the cross-cutting issues (p<0.0001) but rights-based care standards did not change significantly (p=0.2). Similarly, the largest improvement in Ghana was around the cross-cutting issues, that is, 18% (p=0.001). The 10% change in clinical care standards was not statistically significant (p=0.12). In Tanzania, only the 19% increase in rights-based care reached statistical significance (p=0.012).

### Clinical care for women and newborns: EMEN standards 1–3

Clinician observations (table 4) showed that, in Bangladesh, while no labour was monitored on a partograph at baseline, 32 (34%) were monitored in EMEN-QI facilities compared with 9% in comparison facilities in 2018. Blood pressure measurement uptake increased from 33% at baseline to 95% in 2018. Urine testing for pre(eclampsia) screening was still poorly done (1%). Abdominal examination, fetal heart rate (FHR) measurement and active management of the third stage of labour were universally done in Ghanaian EMEN-QI facilities by 2018. At baseline, 17% (13) women had their urine tested proteins, increasing 3.4 folds to 58% (89) in 2018. In Tanzania, urine testing increased from 5% (3) to 32% (22) in EMEN-QI facilities.

**Table 4:** Clinical assessors’ observation of critical intrapartum care indicators for women with complete data from labour to delivery by country

Care component observed in assessment	EMEN intervention facilities			Comparison facilities		
Bangladesh	Pre (153)	Post (178)	Difference	Pre (164)	Post (259)	(±) Difference
Labour monitored on a partograph	0 (0.0%)	32 (33.7%)*	+33.7%	0 (0.0%)	13 (9.4%)*	+9.4%
Woman’s blood pressure measured	51 (33.3%)	169 (94.9%)	+61.6%	115 (70.1%)	186 (71.8%)	+1.7%
Urine tested	2 (1.3%)	2 (1.1%)	−0.2%	1 (0.6%)	4 (1.5%)	+0.9%
Abdominal examination done	74 (48.4%)	154 (86.5%)	+38.1%	80 (48.8%)	154 (59.5%)	+10.7%
Fetal heart rate auscultated	14 (9.2%)	140 (78.7%)	+69.5%	33 (20.1%)	114 (44.0%)	+23.9%
Active management of 3rd stage of labour	99 (77.3%)†	82 (77.4%)†	+0.1%	152 (89.9%)†	101 (70.6%)†	−19.3%
Newborn assessed to be assisted to breathe	119 (97.5%)^¥^	79 (73.1%)^¥^	−22.4%	140 (84.8%)^¥^	122 (89.7%)^¥^	+4.9%
Ghana	Pre (76)	Post (154)		Pre (58)	Post (130)	
Labour monitored on a partograph	49 (64.5%)	113 (75.3%)‡	+10.8%	43 (74.1%)	87 (68.5%)‡	−8.6%
Woman’s blood pressure measured	72 (94.7%)	135 (88.8%)§	−5.9%	52 (91.2%)¶	116 (89.9%)**	−1.3%
Urine tested	13 (17.1%)	89 (58.2%)	+41.1%	14 (24.6%)¶	11 (8.5%)**	−16.1%
Abdominal examination done	74 (97.4%)	154 (100%)	+2.6%	53 (93.0%)	113 (86.9%)	−6.1%
Fetal heart rate auscultated	74 (97.4%)	154 (100%)	+2.6%	54 (94.7%)	123 (94.6%)	−0.1%
Active management of 3rd stage of labour	72 (96.0%)^∞^	147 (96.7%)^∞^	+0.7%	52 (94.5%)^∞^	121 (96.0%)^∞8^	+1.5%
Newborn assessed to be assisted to breathe	66 (88.0%)^#^	148 (99.3%)^#^	+11.3%	52 (94.5%)^#^	123 (95.3%)^#^	+0.8%
Tanzania	Pre (65)	Post (68)		Pre (55)	Post (59)	
Labour monitored on a partograph	65 (100%)	62 (91.2%)	−8.8%	55 (100%)	46 (78.0%)	−12.0%
Woman’s blood pressure measured	49 (75.4%)	56 (82.4%)	+7.0%	55 (100%)	43 (72.9%)	−27.1%
Urine tested	3 (4.6%)	22 (32.4%)	+27.8%	5 (9.1%)	14 (23.7%)	+14.6%
Abdominal examination done	57 (87.7%)	60 (88.2%)	+0.5%	45 (81.8%)	46 (78.0%)	−3.8%
Fetal heart rate auscultated	64 (98.5%)	57 (83.8%)	−14.7%	55 (100%)	46 (78.0%)	−12.0%
Active management of 3rd stage of labour	56 (94.9%)††	44 (100%)	+5.1%	51 (100%)††	40 (100%)	0%
Newborn assessed to be assisted to breathe	57 (87.7%)	47 (69.1%)	−18.6%	51 (92.7%)	38 (64.4%)	−28.3%

* Data for only 95 intervention and 138 comparison participants.

† Data missing for four participants.

‡ Data missing for 3 participants.

§ Data for 152 participants.

¶ Data missing for one participant.

** Data missing for 1 participant.

†† Data for 51 intervention and 59 comparison participants at baseline.

‡‡ Data for 153 participants.

**Figure 3:**
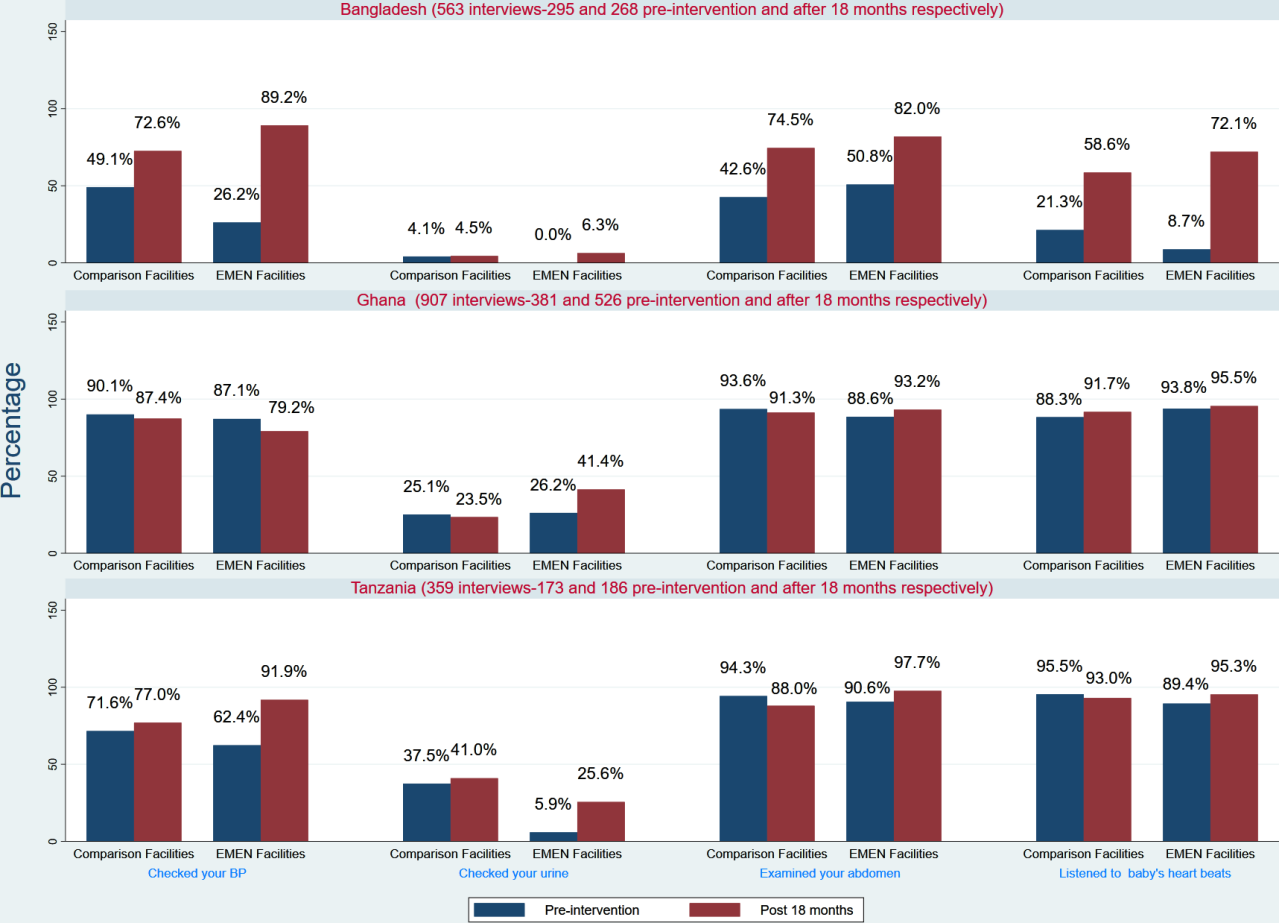
Experiences of clinical care around childbirth by women in the three countries. EMEN, Every Mother Every Newborn.

Figure 3 shows women’s reported experience of clinical care. In 2018, 99 (89%) women in Bangladesh EMEN facilities reported that their BPs were checked at presentation—a significant 63% (95% CI=53% to 73%) increase over the baseline (p<0·001). Urine testing for proteins in comparison facilities was unchanged by 2018 (4% vs 5%) but in EMEN-QI facilities, testing increased to 6% in 2018 from 0% at baseline. Women’s reports corroborated clinician observations in Ghana and Tanzania. In both countries, higher percentages of women were assessed for all the clinical modalities and marginal changes occurred at follow-on. The exception was urine testing for proteins for which, in Ghana, women reported significant 15% increase (95% CI=7% to 23%; p=0.0005) from the 26% at baseline with no change in comparison facilities (25% vs 24%). In Tanzania, at follow-on, reported urine testing increased fourfold to 26% in EMEN-QI facilities.

### Patients’ rights: (provision and experience of) respectful maternity care: EMEN standard 4

Clinician observations found improvements in the provision of respectful maternity care (RMC) especially in Ghana and Bangladesh but to a less extent in Tanzania (table 5).

**Table 5:** Respectful maternity care experiences (patients’ rights) from exit interviews and clinician observations of provider–client interactions

Bangladesh	Intervention			Comparison		
	Baseline (105)	Follow-on (94)	Difference	Baseline (136)	Follow-on (137)	Difference
Courteous communication during care giving	55 (52.4%)	57 (60.6%)	+8.2%	93 (68.4%)	84 (61.3%)	−7.1%
Delivery plan communicated	91 (86.7%)	59 (62.8%)	−23.9%	74 (54.4%)	102 (74.5%)	+20.1%
Care withheld due to inability to make informal payments	1 (1.0%)	0 (0.0%)	−1.0%	1 (0.7%)	4 (2.9%)	+2.2%
Reassures or encourages women to allay their fears	–	68 (72.3%)	–	–	87 (63.5%)	

Ghana	Baseline (76)	Follow-on (157)	Difference	Baseline (58)	Follow-on (131)	Difference
Courteous communication during care giving	70 (92.1%)	118 (75.2%)	−16.9%	49 (84.5%)	97 (74.1%)	−10.4%
Delivery plan communicated	40 (52.6%)	151 (96.1%)	+43.5%	38 (66.1%)	109 (83.1%)	+17.0%
Care withheld due to inability to make informal payments	0 (0.0%)	0 (0.0%)	0.0%	0 (0.0%)	2 (1.5%)	−1.5%
Reassures or encourages women to allay their fears	59 (77.6%)	147 (93.6%)	+16.0%	40 (69.0%)	121 (92.4%)	+23.4%

Tanzania	Baseline (65)	Follow-on (68)	Difference	Baseline (55)	Follow-on (59)	Difference
Courteous communication during care giving	60 (92.3%)	56 (82.4%)	−9.9%	48 (87.3%)	55 (93.2%)	+5.9%
Delivery plan communicated	51 (78.5%)	46 (67.6%)	−10.9%	34 (61.8%)	35 (59.3%)	−2.5%
Care withheld due to inability to make informal payments	5 (7.7%)	3 (4.4%)	−3.3%	8 (14.5%)	6 (10.2%)	−4.3%
Reassures or encourages women to allay their fears	53 (81.5%)	42 (61.8%)	−20.1%	33 (60.0%)	45 (76.3%)	+16.3%

In Bangladesh EMEN-QI facilities, 8% more women were communicated to in a courteous manner during maternity care in 2018 compared with baseline. However, this increase resulted in EMEN-QI facilities equalling the 61% that prevailed in comparison facilities, given EMEN-QI facilities were worse than comparison facilities at baseline (52% vs 68%). Health providers in comparison facilities were more likely to communicate their delivery plan to women in labour after they examined them than those in EMEN-QI. Uptake of this communication fell from 87% at baseline to 63% at follow-on in EMEN-QI facilities while it increased from 54% to 75% in the comparison facilities.

In Ghana, whereas 52% of women in EMEN-QI facilities were told the delivery plan at baseline, the practice was almost universal by 2018 (96%). There was a 17% increase in the practice in comparison facilities. Care was never withheld from any woman in EMEN-QI facilities due to inability to pay illegal charges demanded by health workers, but the practice still persisted in comparison facilities. In Tanzania, comparison facilities seemed to have improved better than the EMEN-QI facilities in RMC.

### Cross-cutting issues: EMEN standards 5–10

In Bangladesh, all EMEN-QI facilities had sphygmomanometers, freezers for storage and baby-sized bag and mask by 2018 compared with baseline (online supplemental table S3). Availability of functional weighing scales dropped from 80% to 40%. In Ghana, functional key equipment were universally available in EMEN-QI facilities. In 2018, four (50%) more facilities had radiant warmers than at baseline (88% vs 38%) but one did not have a functional weighing scale. In Tanzania, there was an overall reduction in the percentage of facilities with functional equipment in 2018 compared with baseline except for pulse oximeters (1—17% vs 5—83%).

Eight selected essential drugs that address the top three causes of maternal and newborn deaths were more available in EMEN-QI facilities across all countries than comparison ones (online supplemental table S3). For instance, cephalosporins were universally available in Bangladesh, Ghana and two-thirds of Tanzanian facilities. Corticosteroids were also universally available in Bangladesh facilities at follow-on; 37.5% of Ghanaian and all Tanzanian EMEN-QI facilities did not have corticosteroids at follow-on.

**Figure 4:**
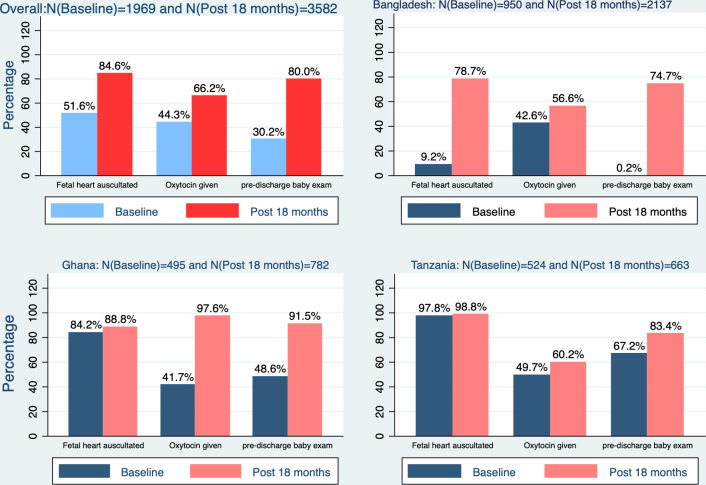
Changes in the documentation of care between baseline (2016) and post 18 months assessment.

Our review of 1969 and 3582 records at baseline and 2018, respectively, showed that documentation of three critical indicators around childbirth increased significantly in EMEN-QI facilities (figure 4). Oxytocin administration for Active Management of the Third Stage of Labour (AMSTL) was documented for 84.6% at follow-on versus 51.6% at baseline and fetal heart auscultation 66.2% vs 44.3%. Bangladesh had the poorest documentation rates at baseline with only 0.2% of newborn exams and 9.2% of FHR auscultation documented. However, at follow-on, these rates increased significantly to over 70%. There were improvements in the use of data for programme and management decision-making.

Availability of HRH was consistently cited as a challenge in all countries. For example, 92 health professionals were posted to Bangladeshi EMEN-QI facilities (18 doctors and 67 nurses/midwives) but just over half of these were available to supervise the 2086 births in 2018. Also, the procurement of life-saving equipment and their functionality was not matched with development of capacity to maintain these: some equipment were poorly maintained and deteriorated over the 18 months of the implementation.

### Sustainability of the interventions

Key informants opined that EMEN-QI aligned with national goals and was sustainable. Sustainability facilitators cited included the collaboration with ministries of health and devolvement of capacity building to districts/facilities. Districts were supported to sustain the gains made in EMEN-QI as QI reports were integrated into routine performance reviews. Threats to sustainability were HRH shortages, high staff attrition without transitional transfer of skills, poorly defined mechanisms for equipment maintenance and weak internal monitoring and evaluation systems. Key informants believed that, EMEN-QI raised staff ‘awareness and consciousness’ on maternal and newborn deaths. In Ghana, a paediatrician singled-out the clinical mentorship model and weekly mortality review conference calls as pivotal to success. Key informants cautioned that the interpretation of the findings should be made with extreme caution as ministries of health in all three countries were phase I countries of Global Quality, Equity Dignity Network and started scaling-up the same standards to non-intervention areas even before EMEN-QI was completed.

## Discussion

Despite initial increases in mortality indicators within the first year of implementation, EMEN-QI achieved a statistically significant 25% reduction in NCFR in Bangladesh (p=0.017) and 85% in Tanzania (p<0.001); 43% reduction in MMR in Ghana (p=0.046) and 41% reduction in PMR in Tanzania (p<0.01). For all mortality indicators, the downward trajectory suggested a positive impact of the EMEN-QI standards implementation on mortality, consistent with recent studies on implementation of QI interventions.^20^,^22^ This study is among the very few that demonstrated statistically significant mortality reductions.^23 24^ Indeed, Walker *et al*^25^ found a 34% reduction in deaths among perinates when they combined QI interventions involving WHO Safebirth Checklist, mentoring and QI collaboratives but their intervention was targeted at babies born preterm or with low birth weight.

Initial increases in mortality indicators could be the result of increased in-referrals to the newly established neonatal care units with consequent increased deaths, and coincided with improved data from the facilities including how they documented care-giving (figure 4). While not attributing the effects solely to EMEN-QI interventions, these mortality reductions seemed logical consequences of improvements in inputs and processes of care. In Tanzania, however, there were only marginal improvements in the indicators to match the mortality reductions achieved. A possible explanation is that QI helped facilities to effectively use the results of the already high uptake of clinical assessments to save lives. The very low NCFR may also indicate residual deficits in the quality of data.

We demonstrated that EMEN-QI (and consequently the WHO) standards are feasible to implement and institutionalise in resource-limited settings: our 39-criteria institutionalisation index score increased from 61% to 80% over the 18-month period, exceeding the EMEN-QI target of 75%. Institutionalisation is facilitated by good leadership with the requisite focus of investment and activities.^26^ In all countries, MPDSR in EMEN-QI facilities induced substantial improvements in case management and fatality rates as audit recommendations fed directly into QI activities.

Our study had some limitations; first, the before-and-after design, despite the inclusion of the comparison facilities, limits the power of attribution of the effects solely to EMEN-QI. Second, the EMEN-QI districts were selected with an equity bias and so deprived, UNICEF-focused districts were used. The findings may therefore not be generalisable to the respective country context and interpretation will require caution. Identifying problems is only one step but providing appropriate and timely response save lives. Our assessment could not measure critical EMEN-QI criteria such as percentage of antenatal corticosteroids correctly given in preterm labour or responses of clinical staff when partographs showed deviations from normal trajectories. Also, observations of client–provider interactions will be subject to the Hawthorne effect as healthcare providers may exaggerate the care they routinely provide. We considered that being there for 2 weeks was enough time for their routine practices to emerge. We could not compare mortality outcomes with those of the comparison facilities due to lack of accurate and reliable routine data.

Notwithstanding, this study had many strengths; it implemented the standards as a package using uniform methods across the three countries. This makes several indicators amenable to cross-country comparisons. It was implemented within programmes, with substantial local input and leadership across the three countries. This has laid critical pathways to sustainability. The implementation was through a south–south collaboration which included MoH, research institutions and development partners in the respective countries with valuable lessons on how these collaborations work to achieve common goals. It was also a large study with this analysis pooling together findings from 43 facilities from the three countries. Furthermore, the use of a variety of methods and their eventual triangulation provided internal validation for the study results.

The effects achieved could have been better if they were not ‘contaminated’ by concurrent implementation of various criteria within EMEN-QI standards in comparison facilities by national governments. For example, in Bangladesh, the quality assurance unit was changed to QI secretariat, and the ‘5Ss’, total quality management and PDCA cycles were introduced in comparison facilities too. Bangladesh demonstrated the largest effects possibly due to interlinking factors: consistent with findings from Winter *et al*’s assessment,^27^ facilities in Bangladesh had the lowest percentage uptake for many of the standards at baseline (46·7% composite index). Second, quality secretariat worked collaboratively with professional bodies in the implementation. Thirdly, very early in the implementation, national stakeholder buy-in was high and the ‘Kurigram experience’ was expected to be the national QI model. This was motivational to districts/facilities. National leadership is credited with these developments.^28^ Similarly, Ghana introduced EMEN-QI criteria into the checklist for facility accreditation within its National Health Insurance Scheme (NHIS). All facilities including comparison ones started implementing EMEN-QI criteria to earn NHIS accreditation as NHIS became the main source of funding for facilities. Tanzania also started a nationwide ‘Star-rating’ system for facility accreditation which included criteria from EMEN-QI standards and so were being universally implemented even in comparison facilities.

Sustainability of such initiatives is key; unless systematically built-in, when the initial investment and intensive efforts of the funders cease, these initiatives collapse.^24 29^ Though the above-mentioned ‘contaminations’ might have diluted the effects of EMEN-QI on mortality outcomes, they are clear indications that the governments of all three countries have adopted the standards—a pathway to sustainability. Lessons from previous initiatives informed our pathways to sustainability: EMEN-QI implementation was therefore done through the ministries of health of the respective countries and included systematic capacity development for sustaining the QI model as a culture in the health service delivery rather than a project. All the planning and budgeting was done in collaboration with the district and facility management and UNICEF provided the funds. Steering committees or teams were formed at the ministerial, regional and district level in each country and these teams were assisted to conduct internal assessment and facilitative supportive supervision. These sustainability models accord with successful models implemented in or recommended from other successful efforts from similar settings.^23 29^ We acknowledge that sustaining and scaling-up such initiatives may still require some continued support to national governments in models similar to UNICEF’s.

There remain some critical gaps in the uptake of quality standards that will require attention. For instance, 66% of women in labour were not monitored on a partograph in Bangladesh after 18 months of implementation (c.f. 100% at baseline). Similarly, in Ghana, urine protein testing to screen for (pre-) eclampsia, the second most important cause of maternal (and fetal) deaths, was not done for 42% of women and for 68% of Tanzanian women. These gaps arise from a combination of lack of HRH with the requisite skills and attitudes to deliver quality and may result in abuses^30 31^ and increased risk of adverse outcomes.^28^ UNICEF/WHO considers having the requisite physical infrastructure, supplies, leadership/governance, and human resources with the knowledge, skills and capacity to address routine and complicated childbirth as a reflection of the robustness of health systems. Quality care is said to be institutionalised only when the uptake of quality standards results in improved childbirth outcomes, reduces or promptly manages complications in a manner that is respectful, discourages mistreatments, supports women and maintains their dignity even after childbirth.^32^,^34^ EMEN-QI was implemented around critical life-saving interventions^35^,^37^ encompassing the seven high priority thematic areas in the vision for the ENAP.^11^ In the process, we learnt that context is important and change that results in mortality reduction takes time.

In conclusion, the EMEN-QI implementation in Bangladesh, Ghana and Tanzania confirmed that the EMEN-QI and WHO MNH quality standards are feasible to implement as a package within health systems in LMICs. It can reduce mortality and improve quality-of-care content and documentation. Modelling the implementation to secure health system buy-in across countries, facilitates progress and lays critical foundations for scale-up and sustainability. Implementation, however, needs to be tailored to the country context.

## Supplementary material

10.1136/bmjgh-2022-009471 online supplemental file 1

## Data Availability

Data are available upon reasonable request.
