# Supplementary material for: Institutionalising maternal and newborn quality-of-care standards in Bangladesh, Ghana and Tanzania: a quasi-experimental study
Source: BMJ Glob Health. 2022 Sep 20;7(9):e009471. doi: 10.1136/bmjgh-2022-009471 (PMC9490604; doi:10.1136/bmjgh-2022-009471)
Supplement: online supplemental file 1 [file bmjgh-2022-009471supp001.docx]

**REFLEXIVITY STATEMENT**

**Study conceptualisation**

*How does this study address local research and policy priorities?*

The manuscript’s background elucidates the fact that the quality of maternal and newborn care provided at health facilities in LMICs has become the missing link between improved process of care and the health outcomes. Quality improvement is a key component of national MNCH priorities across countries to achieve the targets of the global Every Newborn Action Plan (ENAP). EMEN-QI is the strategy for quality improvement in ENAP and therefore, the implementation of the EMEN-QI standards was conceived jointly by the ministries of health in the respective countries, WHO and UNICEF, their partners. Measuring the progress made in implementing EMEN-QI interventions as a package-the objective of this assessment-was led by local research institutions-ICDDR,B, NHRC and NMIMR-together with a consultant (AM) who hails from Ghana and has worked in all three countries. The research institutions were encouraged to include nationally relevant research questions in their assessment to respond to specific local priorities.

*How were local researchers involved in study design?*

Researchers from local institutions including the Ministry of Health and nationally recognised independent research institutions (as mentioned above) were directly involved from the conceptualisation of the study, the design, data collection, analysis up to the finalisation of this manuscript.

**Research management**

*How has funding been used to support the local research team(s)?*

Funding for the study were provided directly to the local research institutions. The support included acquisition of various tools for the assessment and to develop capacity within the teams. Local research teams were facilitated to participate and present their work at international MNCH stakeholder forums locally and internationally. The coordinator of the study was also supported to provide capacity strengthening to members of the local research teams including for qualitative data collection and analyses.

**Data acquisition and analysis**

*How are research staff who conducted data collection acknowledged?*

The leadership and members of the local research teams who were involved in the data collection are co-authors on this manuscript. The staff who conducted the actual data collection have also been acknowledged in the report and at various dissemination forums organised for stakeholders.

*How have members of the research partnership been provided with access to study data?*

All the data collected in the study are owned by the local research institutions. UNICEF’s role is to ensure that joint publications such as this manuscript are well coordinated without conflicts. As evidenced from many publications from the individual countries, they have the right to conduct any analysis and author manuscripts to address local and global needs as their local data may provide for and they are being supported to conduct cross-site analysis on some of the outcomes.

*How were data used to develop analytical skills within the partnership?*

Data analysis meetings were held with the teams within the partnership. At these virtual meetings, local researchers were supported to analyse their own data and to support the collation of data across sites. The lead investigators in the respective countries therefore played lead roles in the analysis under the leadership of the lead author (AM).

**Data interpretation**

*How have research partners collaborated in interpreting study data?*

All interpretation of the data were made to be context-specific and context-relevant. After the results were collated, the lead author, with support from UNICEF, coordinated meetings to interpret the findings and provide derivatives (such as briefs) for the respective countries.

**Drafting and revising for intellectual content**

*How were research partners supported to develop writing skills?*

All the research teams were involved in the writing of the manuscript. Many of them were already experienced researchers with excellent writing skills (criteria for their selection) and so several drafts of the manuscripts including responses to reviewer comments had substantial inputs from the research partners.

*How will research products be shared to address local needs?*

Locally relevant policy briefs have been generated from the findings of this study and shared with the respective countries. Dissemination meetings for these findings will be held after this paper is published to provide evidence in support of the national initiatives around quality of maternal and newborn care.

**Authorship**

*How is the leadership, contribution and ownership of this work by LMIC researchers recognised within the authorship?*

All country partners are co-authors on this manuscript. The lead author was so chosen to be one who has experience in all three countries and who hails from one of the countries. The authorship arrangement was agreed by consensus from all the members of the partnership with preference for the lead authorship being site-neutral but coming from an LMIC.

*How have early career researchers across the partnership been included within the authorship team?*

In the authorship, all researchers whose roles qualified them to be authors on the study have been recommended by the local teams and included in the list as co-authors.

*How has gender balance been addressed within the authorship?*

The authorship was agreed on the principle of equity and merit. All qualifying authors were included without prejudice to their gender or other special characteristics.

**Training**

*How has the project contributed to training of LMIC researchers?*

As illustrated above, capacity development initiatives were built into the implementation of the study and local researchers were prioritised in all opportunities for capacity development including virtual workshops and international meetings.

**Infrastructure**

*How has the project contributed to improvements in local infrastructure?*

The project resulted in substantial improvements in the infrastructure in the respective countries. As evidenced from the manuscript, this ranged from support for equipment maintenance to the construction of new health facilities in some countries.

**Governance**

*What safeguarding procedures were used to protect local study participants and researchers?*

Safeguarding of the study participants and researchers were key considerations in the study. For instance, during observations of care, culture and gender sensitivity were considered. Facility care providers were pre-informed and consented to be observed as were the women or caregivers. In the exit interviews with caregivers, privacy (visual and auditory) as well as confidentiality of the data and its collection were ensured. Researchers were also trained on safety procedures and ethics to ensure their own safety and those of their respondents.
